# Development and Validation of a HPLC Method to Determine Griseofulvin in Rat Plasma: Application to Pharmacokinetic Studies

**DOI:** 10.4137/aci.s953

**Published:** 2008-08-27

**Authors:** Bo Wei, Dong Liang, Theodore R. Bates

**Affiliations:** Department of Pharmaceutical Sciences, College of Pharmacy and Health Sciences, Texas Southern University, 3100 Cleburne Street, Houston, TX 77004

**Keywords:** griseofulvin, HPLC, fluorescence, pharmacokinetics, rat plasma

## Abstract

A simple, specific, sensitive, and rapid high performance liquid chromatography (HPLC) method for the determination of griseofulvin in small volumes of rat plasma was developed and validated using warfarin as an internal standard. Biological sample preparation involved simple extraction with acetonitrile, followed by dilution with aqueous mobile phase buffer (20 mM sodium dihydrogen phosphate, pH 3.5) to eliminate any chromatographic solvent effects. Griseofulvin and warfarin were baseline separated and quantitated on a C_18_ reversed phase column (4.6 × 150 mm, 3.5 μm), using a mobile phase composed of a 20 mM aqueous solution of sodium dihydrogen phosphate-acetonitrile (55:45, v/v, pH 3.5) delivered at a flow rate of 1.0 mL/min, and with fluorescence detection (λexcitation = 300 nm, λ_emission_ = 418 nm). The method was proven to be linear over a plasma griseofulvin concentration range of 10 to 2500 ng/mL with a mean correlation coefficient of 0.9996. The intra-day and inter-day accuracy (relative error) were in the range of 0.89% to 9.26% and 0.71% to 7.68%, respectively. The within-day precision (coefficient of variation) was less than 3.0% and the between-day precision was less than 7.5%. The mean recovery of griseofulvin from rat plasma was found to be 99.2%. The limit of detection (LOD) and the limit of quantification (LOQ) of griseofulvin were determined to be 1 ng/mL and 10 ng/mL, respectively. The developed method was successfully applied to quantitatively assess the pharmacokinetics of griseofulvin in rats following a single 50 mg/kg oral dose of the drug.

## Introduction

**Figure 1.**
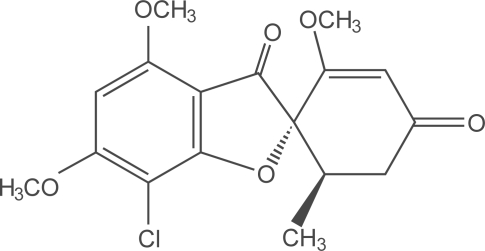
Chemical structure of griseofulvin (C_17_ H_17_ ClO_6_).

Griseofulvin (7-chloro-2’, 4, 6-trimethoxy-6’-methyl-gris-2’-en-3, 4’-dione; Fig. 1) is an oral active antifungal antibiotic derived from the mold *Penicillium griseofulvum* that is primarily used to treat dermatophyte infections in humans and animals. Griseofulvin is a poorly water-soluble drug, which displays a dissolution rate-limited absorption pattern in humans and animals. Hence, it is often used as a model drug to assess the influence of various physicochemical, physiological, and dosage form factors on the absorption kinetics and bioavailability of hydrophobic drugs.

A number of analytical methods have been described for the detection of griseofulvin in biological fluids (e.g. spectrofluorometry [1, 2], gas chromatography (GC) [3–5], thin-layer chromatography [6], high performance liquid chromatography (HPLC) [6–10], and liquid chromatography/tandem mass spectrometry (LC-MS/MS) [11]). Reported spectrofluorometric methods [1, 2] are sensitive, but lack the required specificity. GC methods [3–5] with electron capture detection showed high sensitivity and specificity, but require a derivatization procedure and sample preparation is lengthy. Previously developed HPLC methods with fluorescence detection have such disadvantages as low selectivity [8], large sample size volume [9], and lack of an internal standard [6, 7, 10]. The recently reported electrospray ionization LC-MS/MS method [11] for griseofulvin in human plasma has high specificity and sensitivity, but the instrumentation is expensive, and it requires solid phase extraction of the drug. In addition, there is a paucity of information in the literature on the analysis of griseofulvin in rat plasma with requisite sensitivity and selectivity for preclinical pharmacokinetic studies.

The present report describes a simple, specific, sensitive, and rapid HPLC method with fluorescence detection to determine griseofulvin in small volumes of rat plasma. The method involves a single step acetonitrile extraction of griseofulvin from rat plasma. The limits of detection (LOD) and quantification (LOQ) are greatly improved compared to other reported HPLC methods for griseofulvin in biological media [7–10] and are comparable to the LC/MS/MS method of Mistri and coworkers [11].

## Experimental

### Chemicals and Reagents

Griseofulvin and warfarin sodium, were purchased from Sigma (St. Louis, MO, U.S.A). Acetonitrile (HPLC grade), phosphoric acid (85%, HPLC grade), and sodium dihydrogen phosphate monohydrate (reagent grade) were obtained from Fisher Scientific (Fair Lawn, NJ, U.S.A). All reagents were used as received. Double deionized water was generated by a Milli-Q^®^ academic ultra-pure water purification system (Millipore, Bedford, MA, U.S.A).

### Preparation of Standards and Plasma Samples

Stock solutions of griseofulvin (200 μg/mL) and the internal standard (IS) warfarin (200 μg/mL) were individually prepared in acetonitrile. Ten working standard solutions containing 0.10, 0.25, 0.50, 1.0, 2.5, 5.0, 10.0, 15.0, 20.0, 25.0 μg/mL of griseofulvin were prepared by further dilution of the griseofulvin stock solution with appropriate volumes of acetonitrile. A working solution of the internal standard was prepared by diluting the stock solution of warfarin with acetonitrile to give a final concentration of 50 μg/mL. All stock solutions were stored at 4 °C and all working solutions were freshly prepared daily.

Blank plasma was obtained from untreated rats and spiked with griseofulvin standard solutions to yield calibration standards in plasma at concentrations of 10, 25, 50, 100, 250, 500, 1000, 1500, 2000, 2500 ng/mL. Mobile phase buffer (0.02 M aqueous solution of sodium dihydrogen phosphate, adjusted to pH 3.5 with phosphoric acid) was spiked with griseofulvin standard solutions to obtain calibration standards in buffer at concentrations of 10, 25, 50, 100, 250, 500, 1000, 1500, 2000, 2500 ng/mL. Quality control (QC) standards (n = 8) were also prepared by spiking blank plasma with appropriate volumes of griseofulvin standard solutions to make low, medium and high drug concentrations of 50, 750, 1500 ng/mL, respectively.

Each griseofulvin-containing rat plasma sample (100 μL) was extracted and deproteinized by mixing it with 100 μL of IS working solution containing 50 μg of warfarin per mL of acetonitrile. The mixture was briefly vortex-mixed for 10 sec and centrifuged at 13,000 rpm for 4 min. A 100 μL aliquot of the supernatant was subsequently diluted with 100 μL of mobile phase buffer. The resultant solution was then vortex-mixed for 10 sec, centrifuged at 13,000 rpm for 4 min, and a 50 μL aliquot of the supernatant was directly injected onto the HPLC column.

### Chromatographic Conditions

Chromatographic separations were performed using isocratic elutions at ambient temperature. The Waters HPLC system (Waters Corporation, Milford, MA, U.S.A) consisted of a model 515 pump, a model 717 plus autosampler, a model 474 scanning fluorescence detector, and a computer running Waters Empower 3.2 software. The mobile phase was composed of a mixture of 20 mM aqueous sodium dihydrogen phosphate solution and acetonitrile (55:45, v/v). The final pH of the mobile phase was adjusted to 3.5 with phosphoric acid. The mobile phase was filtered through a 0.45 μm GHP membrane filter (Waters Corporation, Milford, MA, U.S.A) and degassed before use. Griseofulvin and the IS were separated on a reversed phase XTerra^®^ MS C_18_ column (4.6 × 150 mm, 3.5 μm) equipped with an XTerra^®^ MS C_18_ guard column (3.9 × 20 mm, 3.5 μm). The flow rate was set at 1.0 mL/min and the injection volume was 50 μL. Griseofulvin and IS fluorescence measurements were made at excitation and emission wavelengths of 300 nm and 418 nm, respectively.

### Assay Validation

Linear calibration curves for griseofulvin in rat plasma and in buffer were constructed daily by plotting the peak area ratio of griseofulvin to IS *versus* known griseofulvin plasma or buffer concentrations over the range of 10 to 2500 ng/mL.

To validate the assay, three standard calibration curves of griseofulvin in plasma were constructed on the same day to obtain the within-day variation. Another two standard calibration curves of griseofulvin in plasma were constructed on two separate days to establish the between-day variation. Within-day and between-day accuracy and precision were assessed by analyzing quality control (QC) samples at low, medium and high griseofulvin concentration levels (50, 750, and 1500 ng/mL) in eight replicates on three separate days. Analytical accuracy was expressed as the relative percentage error (R.E.) from the theoretical drug concentrations of QC samples. Analytical precision was reflected by the coefficient of variation (C.V.) associated with the mean observed QC concentration at the low, medium and high griseofulvin concentration levels.

The extraction recovery of griseofulvin from rat plasma was estimated from the percentage ratio of the slope of the calibration curve in plasma to the slope of the calibration curve in buffer (n = 3). The limit of detection (LOD) was expressed as the plasma concentration that yielded a peak height equal to three times that of baseline noise. The limit of quantification (LOQ) was expressed as the lowest concentration in the linear calibration curve.

The HPLC peak purity of griseofulvin in dosed rat plasma was tested by comparing the fluorescence (F) to UV peak area response ratios (F/UV PARR) observed with *in vivo* griseofulvin-dosed rat plasma samples to the ratios obtained with reference griseofulvin-spiked buffer samples. The excitation and emission wavelengths used for fluorescence detection were 300 nm and 418 nm, respectively, and the UV detection wavelength was 291 nm. The *in vivo* plasma samples were obtained at 60 and 90 min after administration of a 50 mg/kg oral dose of griseofulvin to Sprague-Dawley rats. Any plasma constituent or griseofulvin metabolite that coeluted with griseofulvin in the dosed rat plasma samples would be expected to alter the F/UV PARR from that of the reference griseofulvin-spiked buffer solutions. Differences between any two mean values were statistically evaluated using the Student’s t-test.

### Pharmacokinetic Studies

The applicability of the developed HPLC method for griseofulvin in rat plasma was demonstrated by the results obtained from pharmacokinetic studies conducted in six male Sprague-Dawley rats (250 g–350 g). Jugular-vein cannulated, adult male rats were fasted 20 hr before and for a 12 hr period after griseofulvin dosing. Each rat received an oral dose of 50 mg/kg of griseofulvin as an aqueous suspension containing 10 mg/mL of griseofulvin and 10 mg/mL of polysorbate 80. Water was allowed *ad libitum*. Serial blood samples (0.20 mL) were collected from the jugular vein cannula before and at 0.5, 1, 1.5, 2, 3, 4, 5, 6, 8, 10, 12, 24 hr after drug administration. The blood samples were centrifuged at 13,000 rpm for 4 min, and plasma samples obtained therefrom were stored at −80 °C until analyzed for drug content. Plasma drug concentration-time data were subjected to noncompartmental pharmacokinetic analyses using the nonlinear regression microcomputer program, WinNonlin (version 2.1; Pharsight Corporation, Mountain View, CA, U.S.A).

## Results

### Chromatography

**Figure 2.**
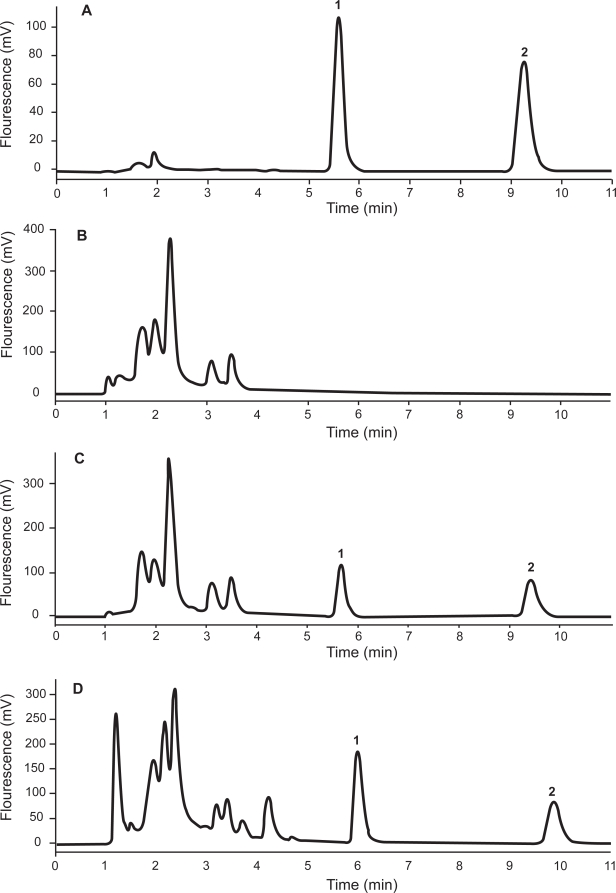

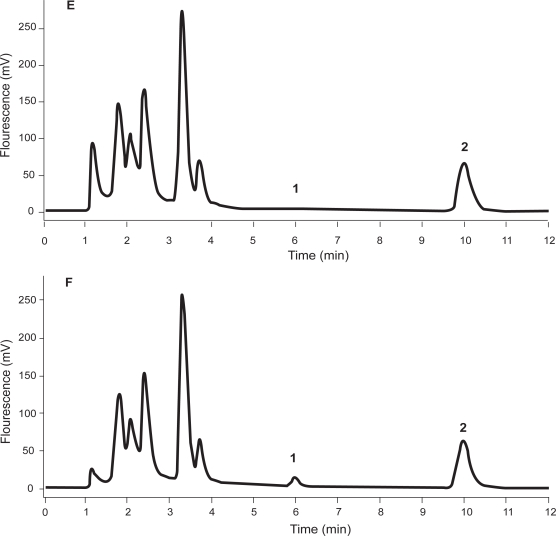
Representative HPLC Chromatograms for (**A**) a standard buffer solution spiked with griseofulvin (500 ng/mL) and the IS (warfarin; 50 μg/mL); (**B**) blank rat plasma; (**C**) blank rat plasma spiked with griseofulvin (500 ng/mL) and the IS (50 μg/mL); (**D**) rat plasma sample obtained 5 hr after a 50 mg/kg oral dose of griseofulvin; (**E**) Limit of detection (LOD) of griseofulvin in rat plasma: 1 ng/mL; and (**F**) Limit of quantification (LOQ) of griseofulvin in rat plasma: 10 ng/mL. Peak 1 = griseofulvin and Peak 2 = IS.

The chromatographic peak for griseofulvin and the IS from an aqueous buffer solution occurred at 5.8 min and 9.4 min, respectively (Fig. 2A). No significant interfering chromatographic peaks appeared for processed blank normal rat plasma samples (Fig. 2B). A processed blank rat plasma sample spiked with griseofulvin (500 ng/mL) and the IS (50 μg/mL) showed baseline separation without any interference from endogenous plasma constituents (Fig. 2C). The chromatogram for a processed plasma sample obtained from a rat 5 hr after receiving a 50 mg/kg oral griseofulvin dose (Fig. 2D) showed a chromatographic separation similar to that of spiked rat plasma (Fig. 2C). The total analysis time for each chromatographic run was 11 min.

The mean F/UV PARR for the griseofulvin orally dosed rat plasma samples (mean ratio ± SD = 76.7 ± 2.9; n = 4) was statistically not different (P > 0.05) from that of the reference griseofulvin-spiked buffer samples (mean ratio + SD = 75.1 + 1.9; n = 6). Thus, the griseofulvin peak obtained with dosed rat plasma that eluted at 5.8 min was pure without any interference from endogenous substances or griseofulvin metabolites.

### Linearity and Range

An excellent linear relationship was established between the griseofulvin to IS peak area ratio and griseofulvin plasma concentration over the concentration range of 10 to 2500 ng/mL. The concentration range chosen for these calibration curves was based on timed plasma griseofulvin concentrations expected in planned *in vivo* oral and intravenous animal pharmacokinetic studies. Five linear calibration curves for griseofulvin in rat plasma were constructed on three separate days [day 1 (n = 3), day 2 (n = 1), and day 3 (n = 1)]. The slope and intercept of each calibration curve was calculated by weighted least squares linear regression, and the mean values yielded the following mean linear equation: y = 0.00202 (± 0.000186)* x + 0.0166 (± 0.0440) [where y = griseofulvin: IS peak area ratio and x = plasma griseofulvin concentration (ng/mL)]. The correlation coefficient for such linear plots ranged from 0.9993 to 0.9998 with a mean value of 0.9996. The within-day and between-day variations in the slope value were small as reflected by coefficients of variation of 2.17% and 2.07%, respectively.

### Accuracy and Precision

The within-day and between-day precision and accuracy of the HPLC assay for griseofulvin in rat plasma were excellent. The intra-day and inter-day accuracy (relative error) were in the range of 0.89% to 9.26% and 0.71% to 7.68%, respectively. The within-day precision (coefficient of variation) was less than 3.0% and the between-day precision was less than 7.5%.

### Limit of Detection and Limit of Quantification

The limit of detection of griseofulvin in rat plasma was 1 ng/mL (Fig. 2E) and the limit of quantification was 10 ng/mL (Fig. 2F).

### Recovery

The mean extraction recovery of griseofulvin from rat plasma was 99.2 ± 0.06% (n = 3), indicating that insignificant amounts of griseofulvin are lost during the plasma protein precipitation step with acetonitrile.

### Application of the HPLC Method to Pharmacokinetic Studies

**Figure 3.**
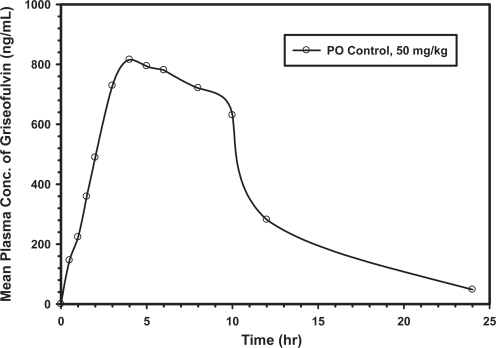
Mean plasma concentration–time profile of griseofulvin (ng/mL) following an oral dose of 50 mg/kg to six Sprague-Dawley rats.

The proposed HPLC method was successfully applied to monitor quantitatively the time course of plasma griseofulvin concentrations after oral administration of a single 50 mg/kg dose of the antibiotic to six adult male Sprague-Dawley rats. The mean plasma drug concentration-time profile observed in these pharmacokinetics studies is shown in Figure 3. The following mean (± SD; n = 6) noncompartmental pharmacokinetic parameters were derived from these data: maximum griseofulvin plasma concentration (C_max_) = 981 ± 308 ng/mL; time of occurrence of C_max_ (T_max_) = 3.33 ± 0.58 hr; total area under the griseofulvin plasma concentration-time curve (AUC) = 9.67 ± 4.9 mg·hr/L; terminal phase elimination half life (T1/2) = 4.30 ± 0.42 hr; apparent volume of distribution divided by the fraction of the oral dose absorbed (Vd/F) = 41.4 ± 22.5 L; total body clearance divided by the fraction of the oral dose absorbed (CL/F) = 6.65 ± 3.35 L/hr; and the mean residence time of griseofulvin in the body (MRT) = 8.14 ± 1.14 hr.

## Discussion

The present HPLC method has several advantages, such as a small sample size requirement, ease of sample preparation, and high sensitivity and selectivity, as compared with previously published chromatography assays [1–10]. Our HPLC method with fluorescence detection has significantly improved selectivity and limit of quantification (10 ng/mL) ten times lower than previously reported HPLC fluorescence assays for griseofulvin in rat plasma [8], which is crucial for pharmacokinetic characterization of drug elimination *in vivo*.

Plasma sample preparation involves single-step extraction of plasma with acetonitrile, followed by centrifugation and dilution of the supernatant with mobile phase buffer before injection onto the HPLC column. We found this buffer dilution step necessary for optimal chromatography.

Our assay with fluorescence detection is comparable to the published LC/MS/MS method [11] in terms of linearity, accuracy and precision, and it has a better rate of drug recovery. Considering the high operational cost and availability of a LC/MS/MS instrument, our HPLC method with fluorescence detection appears best suited for preclinical pharmacokinetic studies.

## Conclusions

A simple, rapid, specific, sensitive and reproducible HPLC method for the quantitative determination of griseofulvin in small volumes of rat plasma has been developed and validated. The method is suitable for studying the oral and intravenous pharmacokinetics of griseofulvin using the rat as an animal model. It is currently being employed in our laboratory to study the effect of simulated weightlessness on the absorption and disposition kinetics of griseofulvin in the well-established tail-suspended rat model for microgravity.
